# Elements of Chemistry

**Published:** 1838-01

**Authors:** 


Art. VIII.—*Elements of Chemistry, including the recent Discoveries and Doctrines of the Science.* By the late Edward Turner, m.d. *Sixth Edition, enlarged and revised.* By Justus Liebig, Professor of Chemistry in the University of Gressen, and Wilton G. Turner.—*London,* 1837. 8vo. Part I. pp. 410.

This is a greatly improved edition of the admirable work of the late accomplished professor of chemistry in University College. It is prepared under the superintendence of the author's brother, and is enriched by much new matter by himself and by the celebrated chemist, Liebig. The latter has undertaken the department of organic chemistry, which has been entirely re-written for the present edition.

